# Immunotherapy in advanced gastroesophageal cancers: A meta‐analysis of sex‐based outcomes in overall survival

**DOI:** 10.1002/ijc.70312

**Published:** 2025-12-24

**Authors:** Michael Masetti, Fausto Petrelli, Filippo Pietrantonio, Sylvie Lorenzen, Alberto Giovanni Leone

**Affiliations:** ^1^ Department of Medicine III (Hematology and Oncology) Klinikum Rechts der Isar, Technische Universität München Munich Germany; ^2^ Oncology Unit, Medical Sciences Department ASST Bergamo Ovest Treviglio Italy; ^3^ Medical Oncology Department Fondazione IRCCS Istituto Nazionale dei Tumori Milan Italy

**Keywords:** gastroesophageal cancer, immunotherapy, sex

## Abstract

Immune‐checkpoint‐inhibition (ICI) represents the mainstay for treatment of advanced gastroesophageal cancer (GEC) in most cases. Sex differences in innate and adaptive immune responses are known to reflect distinct anti‐tumor efficacy of ICI in female and male patients. However, most randomized controlled trials (RCTs) on ICI in GEC were conducted without taking sex differences into account. To address this uncertainty, we conducted a meta‐analysis across first‐line (1L) RCTs in advanced GEC. Systematic research was performed up to March 31, 2025. The primary endpoint was the pooled hazard ratio (HR) for overall survival (OS) in male and female patients, comparing ICI‐based therapy versus control. The secondary objective was to assess the interaction between sex and treatment effect. A total of 15 RCTs were included (7 trials on ESCC and 8 trials on GEA). In male ESCC patients, the pooled HR for OS favored ICI with statistical significance (HR = 0.70, 95% CI = 0.65–0.76, *P* < .00001). In women, HR was 0.81 (95% CI = 0.61–1.07, *P* = .14). In GEA, ICI‐ based therapy showed significant survival benefits in both men (HR = 0.78, 95% CI = 0.73–0.83, *P* < .00001) and women (HR = 0.82, 95% CI = 0.75–0.90, *P* < .0001). The test for interaction between sexes and treatment effect indicated no significant difference in ICI efficacy between sexes in ESCC (Chi^2^ = 1.02, *P* = .31) and GEA (Chi^2^ = 0.78, *P* = .38). In advanced ESCC, only men showed a statistically significant OS benefit from ICI‐based treatment, but the difference between sexes was not statistically significant. In GEA, both sexes had similar survival benefits.

## What's New?

Immune checkpoint inhibitors (ICIs) improve survival in advanced gastroesophageal cancer (GEC), but their benefits may differ by sex. Little is known, however about the impact of sex‐related immune differences on ICI efficacy. In this meta‐analysis, sex‐specific outcomes of ICI therapy were evaluated in advanced GEC, encompassing both esophageal squamous cell carcinoma (ESCC) and gastroesophageal adenocarcinoma (GEA). Data show that in GEA, both men and women experience survival benefits from ICI therapy. Meanwhile, male ESCC patients derive overall survival benefit from ICIs, though apparent sex differences are statistically insignificant. The findings support the inclusion of sex‐specific analyses in future ICI trials.

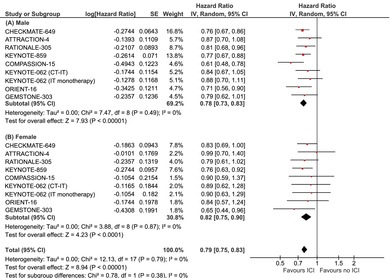

**Abbreviations**
1L: first‐line
Chi^2^ (*χ* ^2^): chi‐squared test
CI: confidence interval
CTLA‐4: cytotoxic T‐lymphocyte‐associated protein 4
EGM: Egger's regression test
ESCC: esophageal squamous cell carcinoma
ESMO: European Society for Medical Oncology
GEA: gastroesophageal adenocarcinoma
GEC: gastroesophageal cancer
HCC: hepatocellular carcinoma
HR: hazard ratio
ICI(s): immune‐checkpoint inhibition/immune checkpoint inhibitor(s)
IPD: individual patient data
NCCN: National Comprehensive Cancer Network
NSCLC: non‐small‐cell lung cancer
OS: overall survival
PD‐1: programmed cell death protein 1
PD‐L1: programmed death‐ligand 1
PRISMA: Preferred Reporting Items for Systematic Reviews and Meta‐Analyses
RCT: randomized controlled trial
SE: standard error
TAM: tumor‐associated macrophage
TMB: tumor mutational burden

## 1 INTRODUCTION

Immune checkpoint inhibitors (ICIs) targeting the programmed cell death protein 1 (PD‐1) or its ligand PD‐L1 represent the current therapeutic mainstay for the treatment of most advanced gastroesophageal cancers (GEC), encompassing both gastroesophageal adenocarcinoma (GEA) and esophageal squamous cell carcinoma (ESCC). ICIs, particularly in combination with chemotherapy, have demonstrated significant survival benefit over chemotherapy alone.^1^, ^2^ This led to regulatory approval of several PD‐1/PD‐L1 targeting agents and recommendation of ICIs as first‐line treatment in international treatment guidelines, such as the European Society for Medical Oncology (ESMO) or US National Comprehensive Cancer Network (NCCN) guidelines.^3^, ^4^

Despite the widespread integration of ICIs in the first‐line treatment setting, emerging evidence suggests that patient‐specific factors, including biological sex, may influence ICI efficacy. Women and men exhibit distinct immunological responses to both foreign and self‐antigens.^5^ Females generally mount stronger innate and adaptive immune responses than men, which results in more rapid clearance of pathogens, explaining the lower severity and prevalence of infections in women and greater response to vaccination than men.^6^, ^7^ In addition, females report higher incidence rates in terms of systemic autoimmune disorders compared to males.^8^

In the immune‐oncology context, several underlying biological factors have been suggested to contribute to sex‐related differences regarding ICI efficacy. The sexual dimorphism of immunity with stronger humoral and cell‐mediated immune responses by females may lead to an increased antitumor activity and susceptibility to immune‐related adverse events.^9^ Conversely, tumors developing in women must bypass stronger immune surveillance and undergo a more intensive immunoediting process to become metastatic. This enhanced ability to evade immune surveillance could make advanced tumors less immunogenic and enriched with stronger immune escape mechanisms. Consequently, advanced cancers in women could exhibit greater resistance to ICI compared to those in men.^10^, ^11^

Another relevant consideration is related to the sexual dimorphism of cancer biology with a disparity of tumor mutational burden (TMB) between sexes. Patients with a high TMB show significant outcome benefit to ICI therapy across various cancer types.^12^ A significantly higher TMB has been reported in male patients' tumors, including melanoma and non‐small‐cell lung cancer (NSCLC), compared to females.^13^, ^14^, ^15^

The sexual dimorphism of hormones and their receptors might also contribute to a sex‐dependent disparity of ICI efficacy. Sex hormones modulate the PD‐1/PD‐L1 signaling pathway and may affect immune function by enhancing PD‐1‐mediated co‐stimulation.^16^ Estradiol, through activation of estrogen receptor α, promotes the polarization of tumor‐associated macrophages (TAMs) toward the immunosuppressive M2 phenotype, while reducing the anti‐tumor M1 phenotype. This shift impairs cytotoxic T cell–mediated antitumor responses and may compromise the efficacy of ICIs in female patients.^17^

Despite the increasing evidence on sex‐based differences in the immune system, studies specifically investigating the interaction between sex and the efficacy of ICI are scarce. As a result, it is still unclear whether there are meaningful sex‐related differences in therapeutic outcomes. Particularly in advanced GEC, the impact of sex on ICI efficacy remains underreported, as randomized controlled trials (RCTs) historically have not prioritized sex‐ and gender‐specific aspects. To address this uncertainty, we investigated the association between sex and outcomes in overall survival (OS) in advanced GEC patients treated with ICIs through a large up‐to‐date meta‐analysis of available RCTs.

## 2 MATERIALS AND METHODS

### 2.1 Search strategy and selection criteria

This meta‐analysis was conducted in accordance with the PRISMA guidelines. A systematic search was performed in PubMed, Embase, and the Web of Science up to March 31, 2025 to identify RCTs investigating immune checkpoint inhibitors (ICIs) in patients with advanced esophageal or gastroesophageal adenocarcinoma (GEA) and squamous cell carcinoma (ESCC). Search terms included combinations of: “*gastric cancer*,” “*(o)esophageal cancer*,” “*gastroesophageal junction*,” “*immunotherapy*,” “*checkpoint inhibitors*,” “*PD‐1*,” “*PD‐L1*,” “*CTLA‐4*,” “*randomized controlled trial*,” “*sex*,” “*gender*,” “*male*,” *and* “*female*.”

### 2.2 Eligibility criteria

Eligible studies were phase II or III RCTs that:

- Compared ICIs (± chemotherapy) with chemotherapy or placebo in the first line;
- Reported hazard ratios (HRs) for OS separately for male and female patients;
- Included adult patients (≥18 years) with histologically confirmed advanced ESCC or GEA.

A summary of the included RCTs is provided in Table S1. Studies focusing exclusively on non‐randomized interventions or those that did not provide sex‐disaggregated outcomes were excluded.

### 2.3 Data extraction and quality assessment

Two independent reviewers extracted data using a standardized form. From each eligible study, the following data were extracted: first author, publication year, study design, treatment *arms*, *number of male and female patients*, *HRs and 95% confidence intervals (CIs) for OS by* sex. When not directly reported, HRs and CIs were estimated from Kaplan–Meier curves or supplementary appendices.

The Cochrane Risk of Bias Tool was used to assess study quality. Discrepancies were resolved by consensus or adjudicated by a third reviewer.

### 2.4 Statistical analysis

The primary endpoint was the pooled HR for OS in male and female patients separately, comparing ICI‐based therapy versus control. A secondary objective was to assess the potential interaction between sex and treatment effect.

Log‐transformed HRs and corresponding standard errors were calculated from the reported 95% CIs. Meta‐analyses were conducted using random‐effects models (DerSimonian and Laird method). Heterogeneity was assessed using the Cochran's *Q* test and quantified with the *I* ^2^ statistic.

A test of subgroup differences (interaction test) was performed to evaluate whether the treatment effect significantly differed between males and females. Forest plots were generated to visualize pooled results. All analyses were performed using RevMan 5.4.

## 3 RESULTS

### 3.1 Study selection and characteristics

**FIGURE 1:**
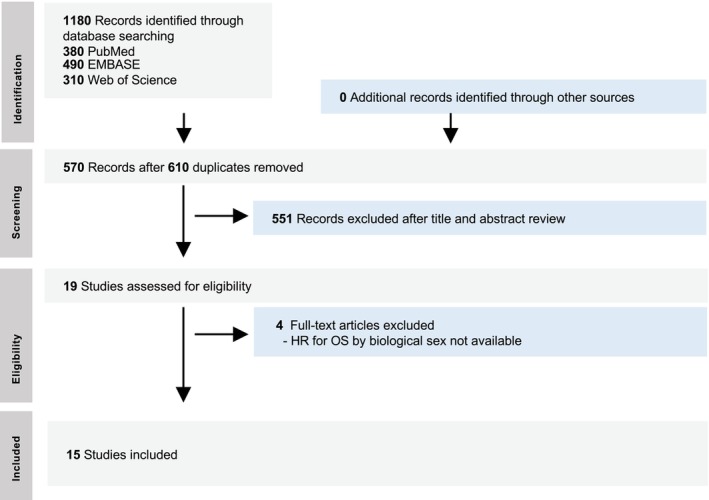
PRISMA flow chart diagram.

A total of 15 RCTs were included in the meta‐analysis, comprising 7 trials on ESCC (RATIONALE‐306, CHECKMATE‐648, ASTRUM‐007, JUPITER‐06, ORIENT‐15, ESCORT‐1ST, GEMSTONE‐304)^18^, ^19^, ^20^, ^21^, ^22^, ^23^, ^24^, ^25^ and 8 trials on GEA (CHECKMATE‐649, ATTRACTION‐4, RATIONALE‐305, KEYNOTE‐859, COMPASSION‐15, KEYNOTE‐062, ORIENT‐16, GEMSTONE‐303).^26^, ^27^, ^28^, ^29^, ^30^, ^31^, ^32^, ^33^ The PRISMA flow diagram detailing study selection is provided in Figure 1. A summary of survival outcomes of the included RCTs is provided in Table 1. Baseline characteristics of included RCTs are summarized in Table 2.

**TABLE 1:** Summary of survival outcomes of included RCTs.

Study	Treatment arms (PD‐(L)1 in bold)	*N* (inc in MA)	PFSl; median (months)	OS; median (months)
ESCC				
**RATIONALE‐306** Xu 2023^18^	**Tislelizumab** plus CTx	652	17.2	8.4
	Placebo plus CTx		10.6	5.7
**CheckMate 648** Kato 2024^19^, ^20^	**Nivolumab** with CTx	970	12.8	5.8
	**Nivolumab** plus **ipilimumab**		12.7	2.9
	CTx (mono)		10.7	5.6
	Placebo plus CTx		9.8	5.8
**ASTRUM‐007** Song 2023^21^	**Serplulimab** with CTx	551	15.3	5.8
	Placebo plus CTx		11.8	5.3
**JUPITER‐06** Wang 2022^22^	**Toripalimab** with CTx	514	17.0	5.7
	Placebo plus CTx		11.0	5.5
**ORIENT‐15** Lu 2022^23^	**Sintilimab** with CTx	659	17.4	7.2
	Placebo plus CTx		12.8	5.7
**ESCORT‐1st** Luo 2021^24^	**Camrelizumab** with CTx	596	15.3	6.9
	Placebo plus CTx		12	5.6
**GEMSTONE‐304** Li 2024^25^	**Sugemalimab** with CTx	540	15.3	6.2
	Placebo plus CTx		11.5	5.4
GEA				
**Checkmate 649** Janjigian 2024^26^	**Nivolumab** plus CTx	1581	7.7	13.8
	CTx (mono)		6.9	11.6
**ATTRACTION‐4** Kang 2022^27^	**Nivolumab** plus CTx	724	10.5	17.5
	Placebo plus CTx		8.3	17.1
**RATIONALE‐305** Qui 2024^28^	**Tislelizumab** plus CTx	997	6.9	15.0
	Placebo plus CTx		6.2	12.9
**KEYNOTE‐859** Rha 2023^29^	**Pembrolizumab** plus CTx	1579	6.9	12.9
	Placebo plus CTx		5.6	11.5
**COMPASSION‐15** Shen 2025^30^	**Cadonilimab** plus CTx	609	7.0	14.1
	Placebo plus CTx		5.3	11.1
**KEYNOTE‐062** Wainberg 2022^31^	**Pembolizumab**	763	2.0^a^	10.6^a^
	**Pembrolizumab** plus CTx		6.9^a^	12.5^a^
	CTx (mono)		6.4^a^	11.1^a^
**ORIENT‐16** Xu 2023^32^	**Sintilimab** plus CTx	650	7.1	15.2
	Placebo plus CTx		5.7	12.3
**GEMSTONE‐303** Zhang 2023^33^	**Sugemalimab** plus CTx	479	7.6^b^	15.6^b^
	Placebo plus CTx		6.1^b^	12.6^b^

Abbreviations: CTx, chemotherapy; ESCC, esophageal squamous cell carcinoma; F, female; GEA, gastroesophageal adenocarcinoma; M, male; MA, meta‐analysis; mono, monotherapy; OS, overall survival; PD‐(L)1, programmed death (ligand) 1; PFS, progression‐free survival.

^a^ PD‐L1 CPS ≥1.

^b^ PD‐L1 CPS ≥.

**TABLE 2:** Summary of baseline characteristics of included RCTs.

RCT	Intervention (*n* patients)	Median age (years)	Sex (%)	Race (%)	ECOG PS (%)	Metastatic disease (%)	PD‐L1 expression (%)	Prior treatment (%)
ESCC								
**RATIONALE‐306** Xu 2023^18^	**Tislelizumab** plus CTx (*n* = 326)	64	M: 87 F: 14	Asian: 74.5 White: 24.2 Other: 1.3	0: 33 1: 67	86	TAP ≥10%: 36 TAP <10%: 46 CPS ≥10: 35 CPS <10: 46	Surgery: 33 Radiotherapy: 12 No therapy: 56
	Placebo plus CTx (*n* = 323)	65	M: 87 F: 13	Asian: 75.2 White: 23.5 Other: 1.3	0: 32 1: 68	87	TAP ≥10%: 33 TAP <10%: 52 CPS ≥10: 35 CPS <10: 50	Surgery: 33 Radiotherapy: 12 No therapy: 56
**CheckMate 648** Kato 2024^19^, ^20^	**Nivolumab** plus CTx (*n* = 321)	64	M: 79 F: 21	Asian: 71 White: 26 Other: 3	0: 47 1: 53	57	PD ≥1%: 49 PD <1%: 51	NR
	**Nivolumab** plus **ipilimumab** (*n* = 325)	63	M: 83 F: 17	Asian: 71 White: 24 Other: 5	0: 46 1: 54	60	PD ≥1%: 49 PD <1%: 51	NR
	CTx (mono) (*n* = 324)	64	M: 85 F: 15	Asian: 70 White: 26 Other: 4	0: 48 1: 52	58	PD ≥1%: 48 PD <1%: 52	NR
**ASTRUM‐007** Song 2023^21^	**Serplulimab** plus CTx (*n* = 368)	64	M: 86 F: 14	NR	0: 251: 75	88	CPS ≥10: 44 CPS <10: 56	NR
	Placebo plus CTx (*n* = 183)	64	M: 84 F: 16	NR	0: 29 1: 71	86	CPS ≥10: 43 CPS <10: 57	NR
**JUPITER‐06** Wang 2022^22^	**Toripalimab** plus CTx (*n* = 257)	63	M: 84 F: 16	Asian: 100	0: 26 1: 74	80	CPS ≥10: 45 CPS <10: 50 CPS ≥1: 78 CPS <1: 17	Radiotherapy: 13.6
	Placebo plus CTx (*n* = 257)	62	M: 86 F: 14	Asian: 100	0: 27 1: 74	77	CPS ≥10: 38 CPS <10: 57 CPS ≥1: 78 CPS <1: 17	Radiotherapy: 13.6
**ORIENT‐15** Lu 2022^23^	**Sintilimab** plus CTx (*n* = 327)	63	M: 85 F: 15	Asian: 98 White: 1 Other: 1	0: 24 1: 76	87	CPS ≥10: 57 CPS <10: 43 CPS ≥1: 90 CPS <1: 10 TPS ≥10%: 36 TPS <10%: 64 TPS ≥1%: 53 TPS <1%: 47	Surgery: 29 Radiotherapy: 17
	Placebo plus CTx (*n* = 332)	63	M: 87 F: 13	Asian: 97 White: 2 Other: 1	0: 76 1: 24	86	CPS ≥10: 58 CPS <10: 42 CPS ≥1: 93 CPS <1: 7 TPS ≥10%: 36 TPS <10%: 64 TPS ≥1%: 57 TPS <1%: 32	Surgery: 34 Radiotherapy: 21
**ESCORT‐1st** Luo 2021^24^	**Camrelizumab** plus CTx (*n* = 298)	62	M: 87 F: 13	NR	0: 24 1: 76	NR	TPS ≥10%: 35 TPS <10%: 63 TPS ≥1%: 56 TPS <1%: 42	Surgery: 39.9 Radiotherapy: 18.1
	Placebo plus CTx (*n* = 298)	62	M: 88 F: 12	NR	0: 22 1: 78	NR	TPS ≥10%: 33 TPS <10%: 65 TPS ≥1%: 55 TPS <1%: 43	Surgery: 33.2 Radiotherapy: 14.1
**GEMSTONE‐304** Li 2024^25^	**Sugemalimab** plus CTx (*n* = 358)	63	M: 88 F: 12	NR	0: 21 1: 79	80	CPS ≥10: 43 CPS ≥1 and <10: 46 CPS <1: 11	NR
	Placebo plus CTx (*n* = 182)		M: 87 F: 13	NR	0: 21 1: 79	79	CPS ≥10: 43 CPS ≥1 and <10: 46 CPS <1: 12	NR
GEA								
**Checkmate 649** Janjigian 2024^26^	**Nivolumab** plus CTx (*n* = 789)	62	M: 68 F: 32	Asian: 24 White: 70 Other: 6	0: 41 1: 59	96	CPS ≥1: 16 CPS <1: 84	Surgery: 20
	CTx (mono) (*n* = 792)	61	M: 71 F: 29	Asian: 24 White: 68 Other: 8	0: 42 1: 57	95	CPS ≥1: 16 CPS <1: 84	Surgery: 22
**ATTRACTION‐4** Kang 2022^27^	**Nivolumab** plus CTx (*n* = 362)	64	M: 70 F: 30	Asian: 100	0: 54 1: 46	NR	CPS ≥1: 16 CPS <1: 84	Surgery: 29 Perioperative CTx: 19
	Placebo plus CTx (*n* = 362)	65	M: 75 F: 25	Asian: 100	0: 54 1: 46	NR	CPS ≥1: 15 CPS <1: 85	Surgery: 29 Perioperative CTx: 16
**RATIONALE‐305** Qui 2024^28^	**Tislelizumab** plus CTx (*n* = 501)	60	M: 69 F: 31	Asian: 75 White: 23 Other: 2	0: 34 1: 66	99	TAP ≥5%: 55 TAP <5%: 45	Surgery: 27 Perioperative CTx: 21
	Placebo plus CTx (*n* = 496)	61	M: 70 F: 30	Asian: 75 White: 22 Other: 3	0: 32 1: 69	99	TAP ≥5%: 55 TAP <5%: 45	Surgery: 28 Perioperative CTx: 20
**KEYNOTE‐859** Rha 2023^29^	**Pembrolizumab** plus CTx (*n* = 790)	61	M: 67 F: 33	Asian: 34 White: 54 Other: 12	0: 36 1: 64	96	CPS ≥10: 35 CPS <10: 64 CPS ≥1: 78 CPS <1: 22	Surgery: 22
	Placebo plus CTx (*n* = 789)	62	M: 69 F: 31	Asian: 34 White: 55 Other: 11	0: 38 1: 62	96	CPS ≥10: 34 CPS <10: 66 CPS ≥1: 78 CPS <1: 22	Surgery: 21
**COMPASSION‐15** Shen 2025^30^	**Cadonilimab** plus CTx (*n* = 305)	64	M: 78 F: 22	NR	0: 23 1: 77	76	CPS ≥5: 38 CPS <5: 52	Surgery: 27 CTx: 15
	Placebo plus CTx (*n* = 304)	64	M: 77 F: 23	NR	0: 24 1: 76	76	CPS ≥5: 46 CPS <5: 48	Surgery: 26 CTx: 14
**KEYNOTE‐062** Wainberg 2022^31^	**Pembolizumab** (*n* = 256)	61	M: 70 F: 30	Asian: 24 White: 58 Other: 18	0: 51 1: 49		CPS ≥10: 36 CPS <10: 64	NR
	**Pembrolizumab** plus CTx (*n* = 257)	62	M: 76 F: 24	Asian: 25 White: 58 Other: 17	0: 46 1: 54	96	CPS ≥10: 39 CPS <10: 61	NR
	CTx (mono) (*n* = 250)	63	M: 72 F: 28	Asian: 24 White: 59 Other: 17	0: 46 1: 54	95	CPS ≥10: 36 CPS <10: 64	NR
**ORIENT‐16** Xu 2023^32^	**Sintilimab** plus CTx (*n* = 327)	59	M: 77 F: 23	NR	0: 27 1: 73	91	CPS ≥5: 60 CPS <5: 40 CPS ≥1: 84 CPS <1: 16 TPS ≥10%: 10 TPS <10%: 90 TPS ≥5%: 15 TPS <5%: 85 TPS ≥1%: 27 TPS <1%: 73	Surgery: 18
	Placebo plus CTx (*n* = 323)		M: 71 F: 29	NR	0: 28 1: 72	93	CPS ≥5: 62 CPS <5: 38 CPS ≥1: 84 CPS <1: 16 TPS ≥10%: 10 TPS <10%: 90 TPS ≥5%: 15 TPS <5%: 85 TPS ≥1%: 23 TPS <1%: 77	Surgery: 18
**GEMSTONE‐303** Zhang 2023^33^	**Sugemalimab** plus CTx (*n* = 241)	63	M: 71 F: 29	Asian:100	0: 26 1: 74	96	CPS 5–9: 46 CPS ≥10: 54	Surgery:25
	Placebo plus CTx (*n* = 238)	63	M: 75 F: 25	Asian:100	0: 23 1: 77	96	CPS 5–9: 46 CPS ≥10: 54	Surgery: 23

Abbreviations: CPS, combined positive score; CTx, chemotherapy; ECOG PS, Eastern Cooperative Oncology Group performance score; ESCC, esophageal squamous cell carcinoma; F, female; GEA, gastroesophageal adenocarcinoma; M, male; NR, not reported; PD‐L1, programmed death‐ligand 1; RCT, randomized controlled trial; TAP, Tumor Area Positivity; TPS, tumor proportion score.

### 3.2 ESCC—Overall survival by sex

**FIGURE 2:**
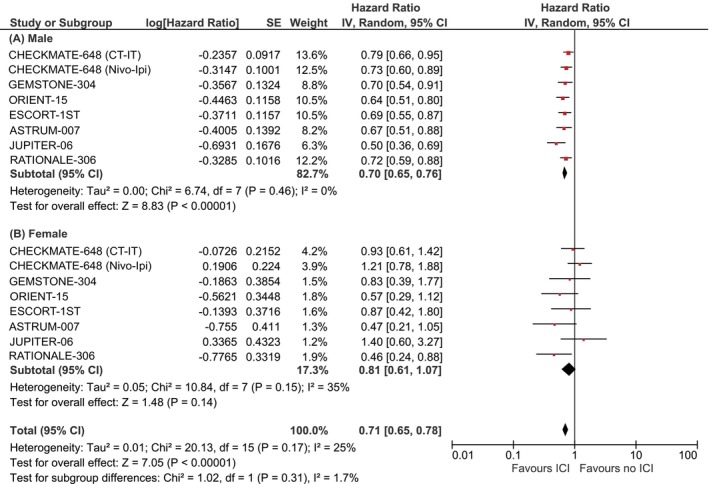
Sex‐dependent overall survival in advanced ESCC. (A) Sex‐dependent overall survival in male patients; (B) Sex‐dependent overall survival in female patients.

In male patients with ESCC (8 subgroups), the pooled hazard ratio (HR) for OS favored ICI‐based therapy with high statistical significance (HR: 0.70, 95% CI: 0.65–0.76, *P* < .00001; Figure 2), and no heterogeneity (*I* ^2^ = 0%). In contrast, in female patients (8 subgroups), the pooled HR was 0.81 (95% CI: 0.61–1.07, *P* = .14), with moderate heterogeneity (*I* ^2^ = 35%). Although a trend toward benefit was observed in females, statistical significance was not reached.

The test for interaction between sex and treatment effect yielded a Chi^2^ = 1.02 (*P* = .31), indicating no significant difference in immunotherapy efficacy between sexes in the ESCC population.

### 3.3 GEA—Overall survival by sex

**FIGURE 3:**
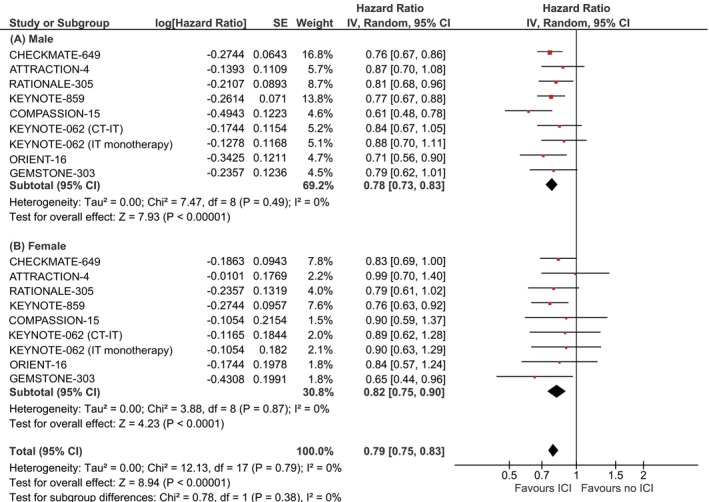
Sex‐dependent overall survival in advanced GEA. (A) Sex‐dependent overall survival in male patients; (B) Sex‐dependent overall survival in female patients.

In patients with gastroesophageal adenocarcinoma, ICI‐based therapy demonstrated significant survival benefits in both sexes. Among males (9 subgroups), the pooled HR for OS was 0.78 (95% CI: 0.73–0.83, *P* < .00001; *I* ^2^ = 0%; Figure 3). Female patients (9 subgroups) also benefited from ICI treatment, with a pooled HR of 0.82 (95% CI: 0.75–0.90, *P* < .0001; *I* ^2^ = 0%).

The test for subgroup differences revealed no significant interaction between sex and treatment effect (Chi^2^ = 0.78, *P* = .38), suggesting that immunotherapy is similarly effective in male and female patients with GEA.

### 3.4 Assessment of publication bias

The presence of publication bias was evaluated using funnel plot asymmetry and Egger's regression test. Funnel plots were constructed by plotting the standard error against the log‐transformed hazard ratios for overall survival from each included study. Visual inspection of the plots revealed no evidence of major asymmetry, with points evenly distributed around the central pooled estimate.

To formally assess small‐study effects, Egger's regression was performed. The analysis regressed standardized effect sizes (log[HR]/SE) against precision (1/SE). The intercept (*B*_0_) represents the degree of asymmetry and potential publication bias.

For the GEA subgroup, the intercept was 0.66 (95% CI: −0.58 to 1.90) with a *t*‐statistic of 1.13 and 16 degrees of freedom, yielding a 1‐tailed *p*‐value of .138 and a 2‐tailed *p*‐value of .277. These results indicate no statistically significant evidence of publication bias.

Similarly, for the ESCC subgroup, the Egger test yielded an intercept of 0.10 (95% CI: −1.31 to 1.52) with a 1‐tailed *p*‐value of .438, supporting the absence of small‐study effects.

Overall, both statistical and visual assessments suggest that publication bias is unlikely to have influenced the results of this meta‐analysis.

## 4 DISCUSSION

To our knowledge, this is the first study to evaluate the efficacy of ICI‐based regimens in advanced GEC according to biological sex. The potential for sex‐based differences in immunotherapy efficacy remains an area of active investigation and debate, and sex‐based differences in ICI response have been reported across various tumor types. A meta‐analysis of eight RCTs evaluating chemo‐immunotherapy in advanced lung cancer demonstrated that women derived a significantly greater benefit from the addition of anti–PD‐1/PD‐L1 agents to chemotherapy compared to men.^34^ Conversely, in patients with advanced or metastatic melanoma and non‐small‐cell lung cancer, men experienced a greater benefit than women when immunotherapy alone was compared to chemotherapy.^35^ More recently, a meta‐analysis of five RCTs investigating ICI‐based regimens in advanced hepatocellular carcinoma (HCC) found that women derived a smaller OS benefit than men, although this difference did not reach statistical significance.^36^

Our results suggest that immunotherapy was associated with a significant OS benefit in male patients with advanced ESCC, but not in female patients, although direct comparison of model estimates revealed no statistically significant difference in treatment effect between sexes. The absence of a significant benefit in female patients may be attributable, on the one hand, to biological differences, and on the other hand, to the limited proportion of female participants in the ESCC trials included in this analysis, which reduced statistical power. While this imbalance is partly driven by the intrinsic male predominance of ESCC—reported to be two to three‐fold more common in males than females globally, though this ratio may be lower or even approach parity in some regions within the ESCC belt—it does not fully explain the disparity.^37^ In the ESCC trials included in this meta‐analysis, the female‐to‐male ratio among enrolled patients was ~1:5, highlighting that the small proportion of women is also a result of their persistent underrepresentation in clinical trials.^38^

With regards to advanced GEA, first‐line immunotherapy‐based regimens were associated with significant survival benefits compared to chemotherapy alone in both sexes, with no apparent difference in the magnitude of benefit. In this context, the male‐to‐female ratio among enrolled patients appears to reflect the actual disease epidemiology, which ranges approximately from 1:2 to 1:3.^39^

Our study has several limitations. First, sex‐disaggregated outcomes were often not primary endpoints of the included trials, and subgroup analyses were underpowered in several studies. This is particularly relevant for female patients with ESCC, where the small sample sizes may hinder the detection of statistically significant differences. Second, lack of individual patient data (IPD) precluded adjustment for potential confounders such as age, performance status, or PD‐L1 expression. These variables are known to influence ICI efficacy, and their omission may have introduced residual confounding that could not be mitigated by study‐level analyses. Third, heterogeneity in trial designs—including differences in chemotherapy backbones, ICI agents, and PD‐L1 testing methodologies—may have introduced variability not fully accounted by our random‐effects model. While the use of random‐effects meta‐analysis mitigates some of this heterogeneity, the influence of unmeasured inter‐trial differences cannot be excluded.

The strength of our study lies in being the first meta‐analysis to examine sex‐based differences in immunotherapy efficacy in GEC, and more broadly, in gastrointestinal cancers.

In conclusion, in advanced ESCC, the OS benefit from first‐line ICI‐based regimens reached statistical significance in male patients only; however, formal comparison of treatment effects between sexes did not reveal a statistically significant difference. Similarly, in advanced GEC, no statistically significant difference in immunotherapy efficacy was observed between males and females, as both sexes appeared to derive comparable survival benefit. These findings not only underscore the importance of considering sex as a biological variable in immunotherapy research, but also stress the urgent need for more equitable sex representation in clinical trials and the availability of individual patient‐level data to allow for more refined, confounder‐adjusted analyses.

## AUTHOR CONTRIBUTIONS

**Michael Masetti:** Conceptualization; investigation; writing – original draft; writing – review and editing; project administration. **Fausto Petrelli:** Formal analysis; data curation; software; validation; methodology; visualization. **Filippo Pietrantonio:** Supervision; writing – review and editing; project administration. **Sylvie Lorenzen:** Supervision; writing – review and editing; project administration. **Alberto Giovanni Leone:** Conceptualization; investigation; writing – original draft; writing – review and editing; project administration.

## FUNDING INFORMATION

This research was supported by Fondazione AIRC associazione Italiana per la Ricerca sul Cancro (IG 2024‐ID. 30422 project – P.I. Pietrantonio Filippo). All other authors have no funding to declare.

## CONFLICT OF INTEREST STATEMENT

FPi reported receiving research funding from Lilly, BMS, Incyte, AstraZeneca, Amgen, Agenus, Rottapharm, Johnson&Johnson, GSK. Personal honoraria from BeOne, Daiichi‐Sankyo, Seagen, Astellas, Ipsen, AstraZeneca, Servier, Bayer, Takeda, Johnson & Johnson, BMS, MSD, Amgen, Merck‐Serono, Pierre‐Fabre, Incyte. Advisory/Consultancy from BMS, MSD, Amgen, Pierre‐Fabre, Johnson & Johnson, Servier, Bayer, Takeda, Astellas, GSK, Daiichi‐Sankyo, Pfizer, BeOne, Jazz Pharmaceuticals, Incyte, Rottapharm, Merck‐Serono, Italfarmaco, Gilead, AstraZeneca, Agenus, Revolution Medicines.

SL reports honoraria from Astellas, MSD, Lilly, BeiGene, AstraZeneca, Servier, Daiichi Sankyo, and BMS; has received research funding from Lilly.

All other authors have no conflict of interest to declare.

## Supporting information


**Table S1.** Overview of included RCTs.

## Data Availability

Data sources and handling of the datasets used in this study are described in the Materials and Methods. Further details and other data that support the findings of this study are available from the corresponding authors upon request.
