# Supplementary material for: Immunotherapy in advanced gastroesophageal cancers: A meta‐analysis of sex‐based outcomes in overall survival
Source: Int J Cancer. 2025 Dec 24;158(10):2697–708. doi: 10.1002/ijc.70312 (PMC12996752; doi:10.1002/ijc.70312)
Supplement: Supplementary file 1 — Table S1. Overview of included RCTs. [file IJC-158-2697-s001.pdf]

Supplementary information

Immunotherapy in advanced gastroesophageal cancers: a meta-analysis of sex-based outcomes in overall survival.

Michael Masetti[1], Fausto Petrelli[2], Filippo Pietrantonio[3], Sylvie Lorenzen[1], Alberto Giovanni Leone[3]

Supplementary Tables

Supplementary table 1: Overview of included RCTs.

Study	Treatment arms (PD-(L)1 in bold)	N (inc in MA)
**ESCC**		
**RATIONALE-306**<br>Xu 2023	Tislelizumab plus CTx<br>Placebo plus CTx	652
**CheckMate 648**<br>Kato 2024	Nivolumab with CTx<br>Nivolumab plus ipilimumab<br>CTx (mono)<br>Placebo plus CTx	970
**ASTRUM-007**<br>Song 2023	Serplulimab with CTx<br>Placebo plus CTx	551
**JUPITER-06**<br>Wang 2022	Toripalimab with CTx<br>Placebo plus CTx	514
**ORIENT-15**<br>Lu 2022	Sintilimab with CTx<br>Placebo plus CTx	659
**ESCORT-1st**<br>Luo 2021	Camrelizumab with CTx<br>Placebo plus CTx	596
**GEMSTONE-304**<br>Li 2024	Sugemalimab with CTx<br>Placebo plus CTx	540
**GEA**		
**Checkmate 649**<br>Janjigian 2024	Nivolumab plus CTx<br>CTx (mono)	1581
**ATTRACTION-4**<br>Kang 2022	Nivolumab plus CTx<br>Placebo plus CTx	724
**RATIONALE-305**<br>Qui 2024	Tislelizumab plus CTx<br>Placebo plus CTx	997
**KEYNOTE-859**<br>Rha 2023	Pembrolizumab plus CTx<br>Placebo plus CTx	1579
**COMPASSION-15**<br>Shen 2025	Cadonilimab plus CTx<br>Placebo plus CTx	609
**KEYNOTE-062**<br>Wainberg 2022	Pembolizumab<br>Pembrolizumab plus CTx<br>CTx (mono)	763
**ORIENT-16**<br>Xu 2023	Sintilimab plus CTx<br>Placebo plus CTx	650
**GEMSTONE-303**<br>Zhang 2023	Sugemalimab plus CTx<br>Placebo plus CTx	479
